# Intelligent penetration testing method for power internet of things systems combining ontology knowledge and reinforcement learning

**DOI:** 10.1371/journal.pone.0323357

**Published:** 2025-05-28

**Authors:** Shoudao Sun, Yi Lu, Di Wu, Guangyan Zhang

**Affiliations:** 1 State Grid Liaoning Electric Power Co., Ltd., Shenyang Power Supply Company, Shenyang, China; Ataturk University, TÜRKIYE

## Abstract

With the application of new-generation information technologies such as big data, artificial intelligence, and the energy Internet in Power Internet of Things (IoT) systems, a large number of IoT terminals, acquisition terminals, and transmission devices have achieved integrated interconnection and comprehensive information interaction. However, this transformation also brings new challenges: the security risk of intrusions into power IoT systems has significantly increased, making the assurance of power system information security a research hotspot. Penetration testing, as an essential means of information security protection, is critical for identifying and fixing security vulnerabilities. Given the complexity of power IoT systems and the limitations of traditional manual testing methods, this paper proposes an automated penetration testing method that combines prior knowledge with deep reinforcement learning. It aims to intelligently explore optimal attack paths under conditions where the system state is unknown. By constructing an ontology knowledge model to fully utilize prior knowledge and introducing an attention mechanism to address the issue of varying state spaces, the efficiency of penetration testing can be improved. Experimental results show that the proposed method effectively optimizes path decision-making for penetration testing, providing support for the security protection of power IoT systems.

## Introduction

The Power Internet of Things (IoT) is the application of IoT technology in smart grids. It effectively integrates power system infrastructure resources by combining various information sensing devices with existing network and database technologies, forming a large intelligent network among electrical devices and between devices and personnel [1]. The rapid development of new-generation information technologies, such as big data, artificial intelligence, and the energy Internet, has propelled the scale of Power IoT information systems into a stage of explosive growth [2]. Leveraging a vast number of IoT terminals, acquisition terminals, transmission devices, etc., it has achieved the integration and interconnection of various types of energy systems, spatiotemporal information, and business information, as well as the perception and information exchange throughout the processes of energy production, transmission, storage, trading, and consumption [3]. Meanwhile, some uncontrollable factors and changes in the physical contact environment have significantly increased the risk of intrusion into power IoT systems. Ensuring the information security of power systems has become a hot research direction in recent years [4, 5].

Power IoT tightly integrates the information systems and physical systems of the power sector, creating an open and interconnected system that significantly increases the information security risks of the entire power system [6]. For instance, attackers can disrupt normal system operations through ransomware attacks, denial-of-service attacks, phishing attacks, malware attacks, zero-day vulnerabilities, and other methods, potentially leading to downtime, equipment damage, triggering emergency responses, and other security incidents. Therefore, to guard against these threats, the power sector needs to formulate and deploy comprehensive security protection measures. Among these, penetration testing is a crucial method for security protection. It helps in identifying and fixing security vulnerabilities, thereby protecting information and business operations from risks such as hacker attacks and data breaches, reducing the likelihood of security incidents [7]. According to MarketsandMarkets [8], the global penetration testing market is estimated to be worth $ 1.7 billion in 2024 and is projected to reach $ 3.9 billion by 2029 at a compound annual growth rate of 17.1% during the forecast period.

During penetration tests, testers employ various attack methods and tools to attempt breaking through the system’s security defenses, thereby identifying and exploiting potential security flaws [6]. Due to the characteristics of vulnerabilities such as their concealment, dynamism, and dependency, penetration testers must not only comprehend the nature of these vulnerabilities within power grid interconnected information systems but also leverage a variety of testing tools to conduct efficient and thorough penetration testing. Therefore, this role is often filled by experts with extensive development experience and comprehensive technical knowledge. The complexity of the power IoT poses significant challenges for manually discovering hidden vulnerabilities, analyzing the attack surfaces, and designing penetration testing schemes [9]. For instance, in a certain power system mini-program, it requires manual packet capturing and data modification several times to uncover an SQL injection method, which can lead to massive data leakage from the database. This highly expert-dependent and labor-intensive testing approach increasingly fails to meet the growing needs of vulnerability penetration testing in the rapid development of the power IoT.

Currently, there are relatively few tools for intelligent penetration testing path planning for the power IoT systems. The primary reason for this gap lies in the lack of effective accumulation and integration of security information specific to power IoT systems. Specifically, while a wealth of useful security information exists—such as vulnerability databases, threat intelligence databases, and security knowledge bases—this knowledge is not fully utilized during penetration testing. On the other hand, reinforcement learning (RL) agents possess the capability for autonomous learning, adjusting and optimizing their strategies based on feedback signals from the environment to find optimal policies. Leveraging RL technology for automated penetration testing of power IoT systems can enable intelligent exploration of target networks, exploitation of potential system vulnerabilities, and automatic generation of optimal attack paths. However, current research on RL-based penetration testing technology primarily focuses on the way of modeling the testing environment and training agents to execute optimal attack path planning strategies. There is relatively less attention given to issues faced during actual penetration testing processes, such as dynamic changes in the state space, incorporation of prior knowledge, and the execution efficiency of models.

To address these challenges, this paper first constructs an ontology knowledge model that describes the state of the system during the penetration testing process, based on prior knowledge about the topology, assets, components, and vulnerabilities of power IoT systems, forming a system state space matrix. We introduce RL theory to iteratively optimize the penetration testing path, exploring optimal path decision-making methods for penetration testing in power IoT systems. Finally, using a distribution automation system as a prototype, we establish a simulation test range to conduct tests, validations, and comparative analyses of the proposed method. The main innovations of this paper are summarized as follows:

1) A penetration testing method for power IoT systems based on deep RL is proposed, which can automatically learn and execute penetration paths even when the system state is unknown;

2) An ontology-based state matrix construction method for the training process of Deep Q-Networks (DQNs) is proposed, leveraging system topology, assets, components, and vulnerability prior knowledge to fully utilize system and security knowledge, thereby avoiding blind exploration behavior during DQN learning;

3) An attention mechanism is adopted to address the issue of changing input state spaces in neural networks, and an optimal path decision-making method for penetration testing is proposed based on the Rainbow DQN framework. The experiment demonstrates that the proposed method can achieve faster convergence with fewer iteration steps across different scenarios.

The subsequent contents of this paper are organized as follows: The Related Works section reviews the current development status of penetration testing technology; The Preliminary section introduces the concepts and theories of deep RL; The Methodology section elaborates on the penetration testing method based on deep RL; The Experiment section describes the experimental methods and results; and the Conclusion section summarizes the entire paper and provides future outlook.

## Related works

Traditional penetration testing primarily relies on manual testing, which must adhere to given security testing methodologies and execution standards. Testers leverage security knowledge bases and the experience of experts to conduct relevant testing work. With the development of artificial intelligence (AI) technology, the processes and methods of penetration testing have become increasingly automated and intelligent. According to a survey by Haq *et al*. [10], as of 2021, using keywords such as “Penetration Testing”, “Pentest” and “Android Penetration”, 1040 relevant publications were identified in databases like IEEE Xplore, ACM Digital Library, and Springer Link, with 380 of these focusing on mobile device penetration testing. Additionally, searching the Engineering Village and Web of Science databases using the keywords “Penetration Testing” and “Security” revealed that over 100 related papers have been published annually since 2019 (based on preliminary screening results). Therefore, penetration testing, as an essential component of information security protection, has remained a key focus of research for security professionals.

Early automatic penetration testing adopted rule-based methods, where a series of actions required for a specific attack tactic are abstracted and then integrated into penetration tools [11]. Common tools include Nmap and Nessus. According to the type of rules, automatic penetration testing can be divided into single-rule and multi-rule categories. Single-rule methods focus on executing a particular attack tactic for a specific task to assist penetration testers in automating targeted penetration tasks but heavily rely on parameter configuration. Typical examples include DCShadow and Atbroker. Multi-rule methods integrate multiple attack tactics to achieve combinational penetration testing, increasing the degree of automation in the testing process [12]. However, the coordination between multiple attack tactics remains relatively low, with the penetration process still depending on parameter configuration. Representative tools include Kaboom, Metasploit, Cobalt Strike, and so on.

The extraction of penetration rules is the most critical requirement in rule-based methods. Depending on the extraction method, it can be categorized into rule extraction based on penetration experience, threat intelligence, and intrusion detection. Literature [13] proposes a semi-automatic knowledge extraction method based on ontology that combines security concepts with security technologies to form a penetration testing knowledge base, enhancing the automation level of penetration testing. Zhou *et al*. used natural language processing techniques to gather intelligence information on advanced persistent threats, combined with regular expressions to extract threat indicators, thereby constructing a penetration testing method based on the ATT&CK matrix [14]. The paper [15] describes an application method for penetration behavior extraction using a security knowledge graph, building a security knowledge graph from audit logs and extracting attack behavior instances using TransE. Rule-based penetration testing relies on rules formed through manual experience and target scenario analysis. While this approach adapts well to the target environment, it heavily depends on expertise and the rule library, making it difficult to generalize to researchers outside this specialized field.

To enhance the intelligence of automated penetration testing, as well as its flexibility and adaptability in complex environments, thereby better serving various testers in need, researchers have recently begun to explore methods that integrate AI technology with penetration testing. These efforts have led to the development of model-based approaches [16]. Reinforcement Learning (RL) based penetration testing is a typical method. It abstracts the penetration testing process into a sequential decision-making process, where the state of the test subject is taken as input, penetration testing actions as output, and the execution results of actions serve as rewards. Through the interaction between the agent and the environment, the agent is trained to converge on the optimal strategy, thereby achieving penetration testing of the environment.

Some RL-based algorithms model the uncertain transition relations of penetration testing states as a partially observable Markov decision process (POMDP) or a Markov Decision Process (MDP), and train through interactions with the environment to develop attack path planning strategies. Sarraute *et al*. [17] pioneered the decomposition of network structures into host-level POMDPs and integrated information gathering into the penetration testing workflow, aligning it with real-world scenarios. Subsequent work by Shmaryahu *et al*. [18] addressed scalability limitations through episodic planning for partial observability. Schwartz *et al*. [19] enhanced realism by modeling defenders’ active responses with information decay factors. POMDP performs excellently in simulating the uncertainty of penetration testing, but its solution complexity becomes significantly high in complex scenarios, making it difficult to apply in large-scale network environments. As a result, an increasing number of researchers have started modeling the penetration testing process as an MDP, which has the advantage of reducing model complexity and improving decision-making efficiency. Yousefi *et al*. [20] used MulVal [21] to generate attack graphs and applied attack graph matrices for MDP modeling. Zennaro *et al*. [22] focused on simplified penetration testing network attack and defense competitions (Capture The Flag) and modeled it as an MDP, solving the problem using a table-based Q-learning algorithm. To mitigate the issue of state and action space explosion, they proposed a method that uses imitation learning to provide prior knowledge for the agent. These methods have successfully modeled the PT problem into the RL paradigm. However, it struggles to mimic complex realistic environments and capture effective features. Observation space’s extension to model the environment further exacerbates computational complexity, limiting fitting performance and speed.

The emergence of DQNs introduces Deep Neural Networks (DNNs) to provide an efficient feature perception, enabling the agent to perceive the environment more efficiently. Zhou *et al*. [23] introduced network information gain as reward signals to guide exploration. Hu *et al*. [24] and Nguyen *et al*. [25] optimized action spaces through graph simplification and multi-level embedding. Sultana *et al*. [26] systematically evaluated deep RL stability across network topologies, revealing critical generalization challenges. These efforts culminated in Zhou *et al*.’s NDSPI-DQN framework [27], which integrated five DQN extensions with action decoupling to reduce dimensionality. Despite progress, unresolved issues in dynamic environment adaptation and invalid action filtering prompted investigations into knowledge-enhanced architectures. The random and ineffective exploration in the early training limits the model’s convergence efficiency and generalization, especially in dynamically and complex environments.

Table 1 provides a comparative analysis of the strengths and weaknesses of rule-based and DQN-based approaches. Rule-based methods rely on predefined logic and features, offering strong interpretability, low false positive rates, and fast execution speed. However, their generalizability is limited, as they can only cover predefined scenarios, and their automation level is relatively low. In contrast, DQN-based methods leverage DNNs to perceive the environment, enabling dynamic adaptation to similar scenarios and fully automated testing, significantly enhancing flexibility and adaptability. However, since these methods rely on data-driven decision-making, they suffer from false positive rates, operate as a “black box” with limited interpretability, and exhibit lower execution efficiency during the exploration phase.

**Table 1:** Comparison of the strengths and weaknesses of different penetration testing techniques.

	Rule-based	DQN-based
Perception Ability	Predefined features	DNN perception
Generalizability	Covers only predefined rule-based scenarios	Adaptive to similar scenarios
False Positive Rate	Low (Explicit rules)	High (Data-dependent)
Interpretability	White-box rules	Black-box process
Automation Level	Semi-automatic	Automatic
Execution Efficiency	Fast rule matching	Ineffective exploration

To address the issues faced by traditional DQN, recent studies emphasized embedding expert knowledge to improve learning efficiency. Zennaro *et al*. [22] simplified penetration testing as MDPs with imitation learning for knowledge injection. Li *et al*. [28] introduced expert prior knowledge to alleviate ineffective exploration, improved learning speed through hierarchical learning. Sychugov *et al*. [29] further explored adversarial inverse reinforcement learning for dynamic networks, introduced tools like “Deep Exploit” for collecting expert data. In [30], the proposed framework encompassed the collection and utilization of expert knowledge. In pretraining phase, the replay buffer primarily incorporates expert knowledge to prevent ineffective exploration. In formal training, the proportion of experience gained through the agent’s exploration is gradually increased in the replay buffer. This approach accelerates model convergence while ensuring generalization capability. Although incorporating prior knowledge into DQN has demonstrated its potential in automated PT, related research remains limited. Moreover, most prior knowledge-based studies have been conducted in known, static testing environments with relatively fixed state spaces. In practical scenarios, an agent may not have complete prior knowledge of the network topology, host assets, and vulnerability information, leading to potential dynamic changes in the state space. This dynamic nature conflicts with the requirement in DQN that the input state space maintains a fixed dimensionality within the neural network structure.

In summary, the automatic and intelligent execution of penetration testing has attracted considerable attention from researchers, leading to numerous theoretical and practical achievements. However, current results still present significant potential for optimization. For instance, addressing the challenge of state space changes due to dynamic or unknown environments, investigating possibilities to enhance DQN techniques to reduce iteration steps for quicker convergence, and whether DQN can handle different scales of test environments when solving the aforementioned issues are topics that require further investigation. Therefore, the subsequent discussion in this paper will delve into these matters.

## Preliminary

### Reinforcement learning

**Fig 1:**
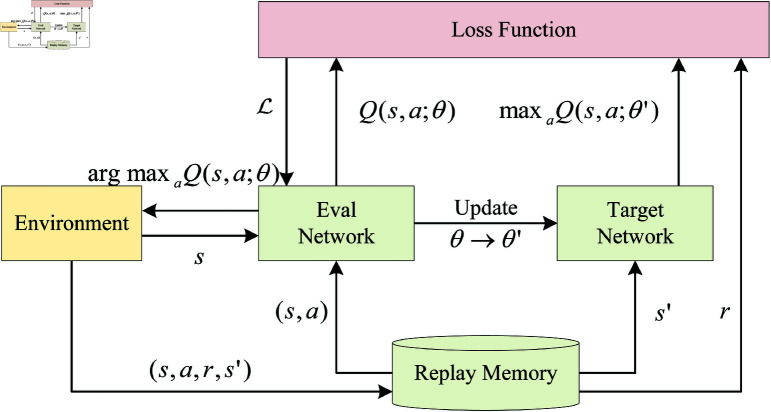
DQN training process.

RL is a variant of machine learning that allows an agent to adjust its behavior by continuously perceiving the state of the environment and the rewards for actions, thereby finding the optimal action strategy in a given scenario [31]. For a standard RL framework, at each time step *t*, the RL agent observes the state $s_{t}\in 𝒮$ from the environment and selects an action $a_{t}\in 𝒜(s_{t})$ according to a policy in the set of policies π. The agent then receives a reward signal $r_{t}\in ℛ$ from the environment and transitions to the next state *s*_*t* + 1_. The goal of the agent is to find the optimal policy $\pi^{*}$ that maximizes the expected cumulative reward:

$$\pi^{*}=\arg\max_{\pi \in \Pi }{𝔼}_{\tau \sim {𝒟}_{\pi }}\left[\sum_{t=0}^{T}\gamma^{t}r_{t}\right]$$ (1)

where γ∈[0,1] is the discount factor determining the importance of future rewards, Π is the set of policies, τ represents the trajectory $\left(s_{0},a_{0},s_{1},a_{1},\ldots ,s_{T}\right)$, and ${𝒟}_{\pi }$ denotes the distribution of trajectories under policy π. Q-Learning is a commonly used technique for finding the optimal policy [32]. For Q-Learning, the Q-function $Q(s_{t},a_{t})$ represents the expected return for taking action *a*_*t*_ in state *s*_*t*_. Its update rule is given by:

$$Q(s_{t},a_{t})\leftarrow Q(s_{t},a_{t})+\varepsilon \left(r_{t}+\gamma \max_{a}Q(s_{t+1},a)-Q(s_{t},a_{t})\right)$$ (2)

where ε∈[0,1] is the learning rate. However, traditional Q-Learning algorithms use tables to store *s*_*t*_ and *a*_*t*_, which poses significant limitations in practice and cannot handle large state spaces. To address these issues, researchers introduced neural networks as function approximators for state-action pairs, leading to deep RL algorithms such as DQN. The training process of the DQN algorithm is illustrated in Fig 1. It introduces two separate networks, the Eval Network and the Target Network, for generating predicted Q-values and target Q-values, respectively. Additionally, it incorporates Experience Replay to improve the efficiency of experience utilization.

The loss function for DQN is defined as:

$${ℒ}_{i}(\theta_{i})={𝔼}_{(s,a,r,s^{\prime })\sim 𝒟}[(r+\gamma \max_{a^{\prime }}Q(s^{\prime },a^{\prime };\theta^{-})-Q(s,a;\theta ){)}^{2}]$$ (3)

where $\theta_{i}$ represents the neural network parameters at the *i*-th iteration, $\theta^{-}$ is the set of parameters for the Target Network, and $r+\gamma \max_{a^{\prime }}Q(s^{\prime },a^{\prime };\theta^{-})$ denotes the Q-value from the Target Network at the *i*-th iteration.

### Rainbow DQN

Rainbow DQN is a comprehensive algorithm that integrates multiple improvements to DQN, first proposed by the DeepMind team in 2018 [33]. It primarily incorporates the following six enhancement strategies:

1) **Double DQN**: By using two networks (target network and behavior network) to estimate the maximum Q-value and select actions separately, it mitigates the overestimation problem of Q-values in DQN [34]. The Q-value calculation for the Target Network in Double DQN is given by:

$$y_{i}=r+\gamma Q(s^{\prime },\arg\max_{a}Q(s^{\prime },a;\theta_{i});\theta^{-})$$ (4)

where $\mathrm{\backslash arg}\max_{a}Q(s^{\prime },a;\theta_{i})$ indicates selecting the best action in the next state using the behavior network $\theta_{i}$, and $Q(s^{\prime },\cdot ;\theta^{-})$ evaluates the value of the selected action using the target network $\theta^{-}$.

2) **Dueling DQN**: This architecture splits into two streams to decompose the Q-value into a value stream *V*(*s*) and an advantage stream A(s,a), better separating state value from action value [35]. The Q-value is computed as:

$$Q(s,a;\theta ,\alpha ,\beta )=V(s;\theta ,\beta )+(A(s,a;\theta ,\alpha )-\frac{1}{\left|A\right|}\sum_{a^{\prime }}A(s,a^{\prime };\theta ,\alpha ))$$ (5)

3) **Prioritized Experience Replay (PER)**: Samples are drawn based on their importance (such as temporal difference error (TD-error)) from the experience replay buffer, prioritizing learning from important samples to accelerate the learning process [36]. The TD-error measures the difference between the current predicted Q-value and the target Q-value, $\delta_{i}=Q(s_{i},a_{i};\theta )$ − *y*_*i*_, and then adds a small constant κ to ensure non-zero priority, $p_{i}=|\delta_{i}|$  +  κ. To sample from the experience replay buffer, each experience’s sampling probability *P*(*i*) is adjusted according to its priority raised to the power ℓ:

$$P(i)=p_{i}^{\ell }/\sum_{k}p_{k}^{\ell }$$ (6)

4) **Multi-step Learning**: By introducing *n*-step returns, it considers the cumulative discounted reward over the next *n* time steps, thus capturing long-term dependencies better [37]. The *n*-step return ${𝒢}_{t}^{\left(n\right)}$ is defined as the sum of immediate rewards from the current step *t* to *t* + *n*−1, plus the maximum expected reward at step *t* + *n*:

$${𝒢}_{t}^{\left(n\right)}=r_{t+1}+\gamma r_{t+2}+\ldots +\gamma^{n-1}r_{t+n}+\gamma^{n}\max_{a^{\prime }}Q(s_{t+n},a^{\prime };\theta^{-})$$ (7)

5) **Noisy Networks**: By introducing parameterized noise sources into network weights, it enables the network to exhibit exploratory behavior naturally across different states [38]. For each weight $\omega_{i}$ in the neural network, the expression with added noise is:

$$\omega_{i}=\mu_{\omega_{i}}+\sigma_{\omega_{i}}\cdot \eta$$ (8)

where $\mu_{\omega_{i}}$ and $\sigma_{\omega_{i}}$ represent the mean and standard deviation of the weight, and η is a noise vector following a certain distribution.

6) **Distributional RL**: Instead of estimating a single Q-value, it aims to learn a probability distribution Z(s,a) describing all possible cumulative discounted rewards, better capturing uncertainty. Categorical DQN is one implementation of this approach [39]. Given the current experience $\left(s,a,r,s^{\prime }\right)$, the goal is to find a new distribution $T_{z}(s^{\prime },a^{\prime })$, calculated as $T_{z}=r+\gamma z_{j}$, and then compute the new distribution $Tp(s^{\prime },a^{\prime })$ based on *T*_*z*_. The loss function typically uses cross-entropy loss:

$${ℒ}_{i}(\theta_{i})=-\sum_{j=1}^{N}Tp_{j}\log p_{j}(s,a;\theta_{i})$$ (9)

## Methodology

### Problem formulation

The problem of automated penetration testing based on DQN can be modeled as an MDP [40], typically represented by the tuple <𝒮,𝒜,ℛ,ℱ,γ>, where: 𝒮 represents the state space of the environment, 𝒜 is the action space, ℛ:𝒮×𝒜↦ℝ denotes the reward function, ℱ:𝒮×𝒜×𝒮↦[0,1] is the state transition probability function, indicating the probability that executing an attack $a_{t}\in 𝒜$ will transfer the state from *s*_*t*_ to *s*_*t* + 1_, γ is the discount factor used to determine the importance of long-term rewards.

1) **State Space** The information acquired by the agent during the penetration process through scanning the environment can be expressed as a state matrix. Each row of this matrix represents the status of each host node in the network and its connections with other hosts, defined as follows:

$$s^{\left(i\right)}=[\{M_{i,j}{\}}_{1\times N},Ass(M_{i}),Attr(M_{i})]$$ (10)

where $\{M_{i,j}{\}}_{1\times N}$ indicates the connection relationship between host node *i* and other nodes in the network, *N* is the network size; *Ass*(*M*_*i*_) and *Attr*(*M*_*i*_) represent the asset information and vulnerability information of host node *i*, respectively. Thus, the entire test environment’s state space is $𝒮=[s^{\left(1\right)},s^{\left(2\right)},\ldots ,s^{\left(N\right)}{]}^{T}$. Subsequent section will detail the method for constructing the state matrix 𝒮.

2) **Action Space** The actions that the agent can execute during the penetration test include a set of behaviors such as collecting asset information (e.g., port scanning, service scanning, operating system (OS) detection, system hardware/software identification), vulnerability scanning (e.g., OS vulnerability scanning, service vulnerability scanning, hardware/software vulnerability probing), and exploitation (specific services based on discovered vulnerabilities). Each action in the action space 𝒜 can be described as a specific attack behavior or tool. It should be noted that the dimensionality of the action space 𝒜 may change as new vulnerabilities are exposed and penetration techniques evolve, leading to an increase in attack tools. If the action space 𝒜 changes, the trained DQN parameters may need to be updated or adjusted, but this is beyond the scope of this paper.

3) **Reward Function** The goal of the agent is to gather additional information about target hosts at minimal attack cost, thereby increasing the success rate of exploiting vulnerabilities to attack the host. Therefore, the Reward Function *r*^(*i*)^ for any host node *i* in the environment can be expressed as:

$$r^{\left(i\right)}=\text{Value}(i)+\text{Score}(v_{i})-\sum_{a\in 𝒜}\text{Cost}(a)$$ (11)

where Value(i) represents the value of host node *i*; $\text{Score}(v_{i})$ is the vulnerability score of host node *i*, which can be calculated using the Common Vulnerability Scoring System (CVSS) formula [41] and the vulnerability information of host node *i*; Cost(a) represents the cost of executing attack *a*, considering factors like execution time and resource consumption.

4) **Optimization Objective** According to Equation (1), the optimization objective for the neural network is to maximize the cumulative reward through executing policy $\pi^{*}$. Therefore, the value function for attack behavior, or Q-function, can be expressed as:

$$Q^{*}(s,a)=\max_{\pi \in \Pi }{𝔼}_{\tau \sim {𝒟}_{\pi }}\left[\sum_{t=0}^{T}\gamma^{t}r(s_{t},a_{t})|s_{t}=s,a_{t}=a\right]$$ (12)

Thus, for a given state *s*, the optimal attack action a^*^ can be obtained as:

$$a^{*}=\arg\max Q^{*}(s,a)$$ (13)

In current state, the system will evaluate all possible actions in the action space to assess their corresponding *Q*-values, determining which action can yield the highest reward. In this selection process, the exploitation of certain vulnerabilities may result in higher *Q*-values, making them more likely to be selected under the effect of the \argmax operation.

### The ontologies of system and security knowledge

**Fig 2:**
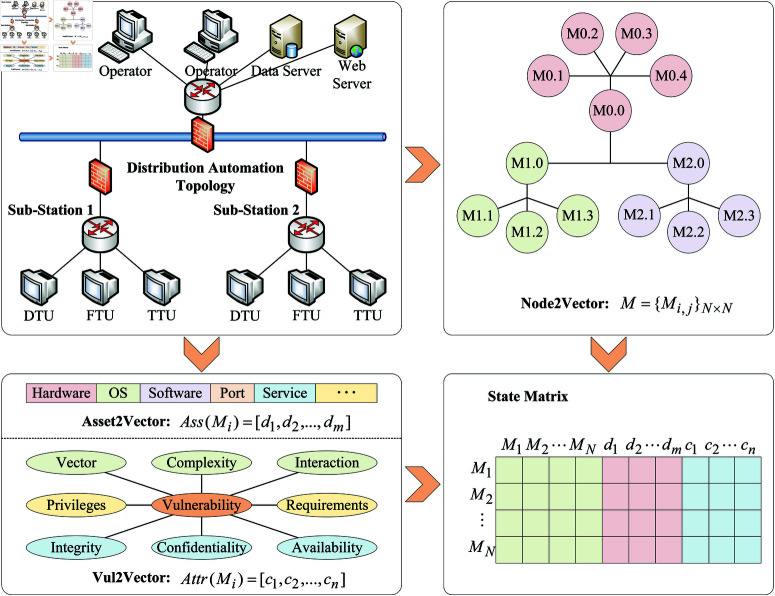
The ontology knowledge.

Compared to allowing the agent to explore blindly, integrating prior knowledge into DQN can significantly enhance the learning capabilities of the agent. Therefore, in this paper, we propose a method that embeds system prior knowledge and security prior knowledge into the DQN learning process, as illustrated in Fig 2.

Firstly, based on the topology of the power IoT (using a distribution automation system as an example in this paper), a Node connection model is established. The connections between nodes are then vectorized (Node2Vector), resulting in a relationship matrix $𝐌=\{M_{i,j}{\}}_{N\times N}$, where *M*_*i*,*j*_ indicates whether there is a connection between node *i* and node *j*: 1 for connected, –1 for not connected, and *N* represents the network scale. Subsequently, each node in the test environment is instantiated according to the ontology knowledge base. The ontology knowledge includes asset attributes of the nodes and vulnerability information. Asset attributes encompass hardware, OS, software, ports, services, etc., while vulnerability information is obtained from the Common Vulnerabilities and Exposures (CVE) vulnerability database and CVSS vulnerability scoring system, providing data on the complexity, exploitability, and associated security risks of vulnerabilities. The asset attributes and vulnerability attributes of the nodes are then vectorized (Vul2Vector and Asset2Vector), yielding asset attribute vectors $Ass(M_{i})=\{d_{1},d_{2},\ldots ,d_{m}\}$ and vulnerability attribute vectors $Attr(M_{i})=\{c_{1},c_{2},\ldots ,c_{n}\}$. Finally, following the approach depicted in Fig 2, the state matrix 𝒮 is constructed.

It is evident that as penetration testing proceeds, the host information, asset information, and vulnerability data of the tested system will be continuously uncovered, leading to an increasingly enriched state matrix. However, it is noteworthy that during penetration testing based on DQN, the Agent can only select actions within its action space. Therefore, if there are no corresponding actions available for newly discovered host asset information and vulnerability data, the Agent cannot exploit these data. Consequently, we can predefine a fixed vector length for asset information and vulnerability data according to the Agent’s action space, ensuring that the dimensions of *Ass*(*M*_*i*_) and *Attr*(*M*_*i*_) remain fixed (the content in the vectors may temporarily be null values). This approach allows us to focus solely on the processing issues of newly discovered hosts (nodes), which will be elaborated in the following sections.

### Rainbow DQN-based modeling method

**Fig 3:**
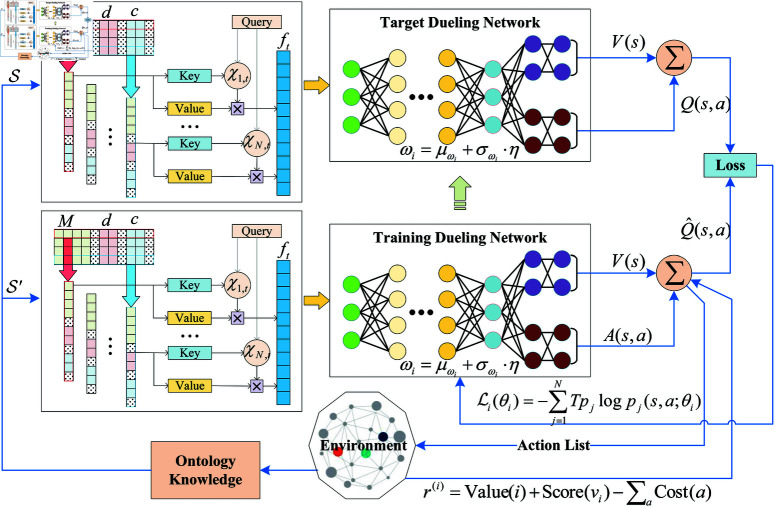
The rainbow DQN-based penetration testing framework.

As mentioned above, the state matrix 𝒮 increases with the number of host nodes explored during the penetration testing process. Consequently, the input dimensions for the DQN network change dynamically. However, a typical DQN employs fully connected layers, which require the number of neurons in each layer to be fixed. To address this issue, this paper introduces an attention mechanism to adapt to the dynamic changes in the state matrix 𝒮, as illustrated in Fig 3.

We decompose the state matrix 𝒮 into two parts. The first part consists of the asset attributes and vulnerability attributes of the host nodes. Since the format of these attributes is fixed, even if a host node lacks a particular attribute, we retain the framework and fill the corresponding section with zeros. Therefore, this part has a fixed dimension, containing elements $\left[d_{1},d_{2},\ldots ,d_{m},c_{1},c_{2},\ldots ,c_{n}\right]$. The second part comprises the connection relationships between host nodes. As the agent explores the network, the number of discovered host nodes increases continuously. For this reason, an attention mechanism is applied to learn from this process. It computes a weighted sum of the relationships to obtain a representation with a fixed dimension:

$$f_{t}(M_{1},M_{2},\ldots ,M_{N})=\sum_{i=1}^{N}\chi_{i,t}\cdot x$$ (14)

where *x* represents the connection relationship of a host node; $\chi_{i,t}$ denotes the attention weight. The calculation of the weight factors can be expressed using the softmax function [42]:

$$\chi_{i,t}=\text{softmax}(\ell_{i,t})=\frac{\exp(\ell_{i,t})}{\sum \exp(\ell_{i,t})},$$ (15)

$$\ell_{i,t}=x_{i}W_{k}^{T}W_{q}x_{i}$$ (16)

where $W_{k}$ and $W_{q}$ are automatically learned parameters, with $W_{k}$ mapping to the Key value of the network and $W_{q}$ mapping to the Query. Through this approach, regardless of the number of host nodes discovered by the agent, the parameters that the DQN network needs to learn remain constant. This not only simplifies the training process but also enhances the agent’s adaptability to dynamic changes in the environment.

**Algorithm 1. Training algorithm of rainbow DQN.**

**Input:** Initial network parameters $\Theta_{\text{train}},\Theta_{\text{target}}$, replay buffer *B*, batch size *m*, learning rate α, discount factor γ, update frequency *F*, and τ for soft update.

**Output:** Trained network $\Theta_{\text{train}}$.

1: Initialize $\Theta_{\text{train}},\Theta_{\text{target}}$ with random weights.

2: Initialize noise parameters for each weight $\mu_{\omega_{i}}$ and $\sigma_{\omega_{i}}$

(mean and standard deviation of the weights).

3: **repeat**

4:   Capture state *s* and connection information from

environment.

5:   Construct connection-state matrix

$𝐙=f_{\text{combine}}(s,\text{connections})$.

6:   Sample ${𝐙}_{\text{sample}}$ from **Z**.

7:   Fuse features into **f** and input into training network

$\Theta_{\text{train}}$.

8:   Apply noisy networks to weights: $\omega_{i}=\mu_{\omega_{i}}+\sigma_{\omega_{i}}\cdot \eta_{i}$.

9:   Compute value *V*(*s*), advantage A(s,a), and Q-values using

dueling architecture.

10:   Apply double Q-learning correction: $\hat{Q}(s,a)=V(s)+A(s,a)$

11:   Select action $a^{*}=\mathrm{\backslash arg}\max_{a}{\hat{Q}}^{\text{(estimate)}}(s,a)$.

12:   Store $\left(s,a^{*},r,s^{\prime }\right)$ in buffer *B*.



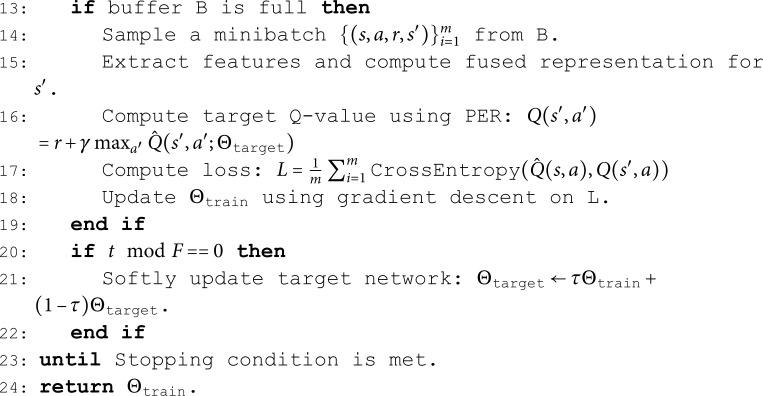



The right-hand side of Fig 3 shows the implementation framework for Rainbow DQN, while Algorithm 1 details the specific training process. Initially, state information *s* is captured from the environment, and a connection-state matrix 𝐙 is constructed. Then, integrated with an attention mechanism, the fused state features are extracted and fed into the Training Dueling Network $\Theta_{\text{train}}$. Next, noisy networks are applied to obtain the weights $\omega_{i}$ of each layer’s connections in the neural network, and the dueling architecture is used to calculate the value stream *V*(*s*) and the advantage stream A(s,a). The Double Q-Learning technique is then applied to correct the Q-value $\hat{Q}(s,a)$, and the action a^*^ that maximizes the Q-value is selected and applied to the target environment. The tuple $\left(s,a^{*},r,s^{\prime }\right)$ is stored in the buffer *B*. This process is repeated until the buffer meets the training requirements. During the training phase, the target Q-values are calculated using Prioritized Experience Replay (PER), and the loss is computed using cross-entropy. Parameters are updated using gradient descent.

## Experiment

### Experimental setup

In this section, we elaborate on several experimental scenarios to verify the effectiveness of our approach by answering the following research questions ( **RQs**).

**RQ1**: Can the proposed method reduce the number of iterations for the agent to achieve faster convergence?

**RQ2**: Is the proposed approach adaptable to rescalable network scenarios?

**Fig 4:**
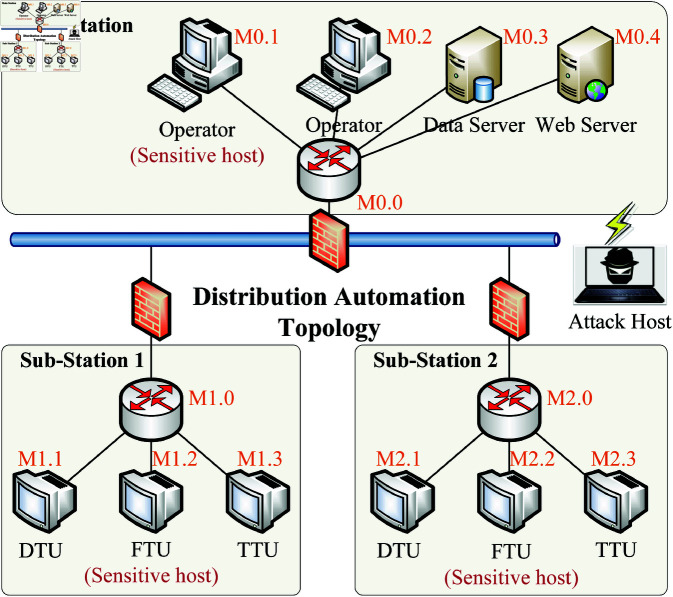
Experimental scenario for penetration testing.

To verify the effectiveness of the proposed method, we established a penetration testing environment for distribution automation systems as shown in Fig 4 using GNS3 (version 2.2.52) and VMware Workstation Pro 17 on a Windows 10 platform. The setup includes one main station and two substations, comprising a total of ten hosts, three subnet switches, and one core switch. Each station contains a sensitive host, which is the target of the penetration test (Operator: M0.1, FTU: M1.2 and M2.2). The configuration information of the hosts in the target environment is summarized in Table 2, where the value of sensitive hosts is set to 100, while non-sensitive hosts have a value of 0. The four hosts in the Main Station run the Windows operating system with services such as HTTP, SSH, FTP, and SAMBA configured; the hosts in the Sub-Stations use Linux OS with services like FTP and SSH. Switches are loaded directly into GNS3 using Cisco images. The attacking host, which serves as the penetration testing host, connects via network bridging to the physical Ethernet interface of the Main Station, aiming to discover the three sensitive hosts within the network and complete the penetration test.

**Table 2:** Configuration information in the experimental platform.

Name	OS	Service	Process	Host-value
M0.1	Windows	HTTP	Schtask	100
M0.2	Windows	HTTP, SSH	Schtask	0
M0.3, M0.4	Windows	HTTP, FTP, SAMBA	Daclsvc	0
M1.1, M1.3, M2.1, M2.3	Linux	SSH	Tomcat	0
M1.2, M2.2	Linux	FTP	Tomcat	100
M0, M0.0, M1.0, M2.0	Cisco	FTP, SSH	–	0

Services and processes frequently exploited by attackers are selected to represent vulnerabilities. For instance, an agent can exploit FTP vulnerabilities to gain Root access on a host or leverage Tomcat vulnerabilities to escalate privileges from User level on a compromised host. Table 3 lists the actions available to the agent within this environment, along with the cost of execution, success probability, and the permission that can be obtained upon successful execution. The action list includes three scanning actions, four service-specific vulnerability exploitation actions, and three process-specific privilege escalation actions. The success probability for vulnerability exploitation actions is set according to CVSS high, medium, and low difficulty levels at 0.2, 0.5, and 0.8 respectively, while the success probability for other actions is set to 1.

**Table 3:** Action list for the agent.

Action	Category	Cost	Probability	Permission
HTTP-Exp	Exploit	2	0.8	User
SSH-Exp	Exploit	3	0.8	User
FTP-Exp	Exploit	1	0.5	Root
SAMBA-Exp	Exploit	2	0.2	Root
Schtask-PE	Promotion	1	1	Root
Daclsvc-PE	Promotion	1	1	Root
Tomcat-PE	Promotion	1	1	Root
Service-Scan	Scan	1	1	-
OS-Scan	Scan	1	1	-
Process-Scan	Scan	1	1	-

The penetration testing agent was developed in a Linux environment using Python 3.9.20, with PyTorch serving as the framework for algorithm implementation. The hardware configuration includes an Intel Core Ultra 7 165H CPU and an NVIDIA A2000 Ada GPU. The training parameters for the model are listed in Table 4.

**Table 4:** List of major parameters in the rainbow DQN.

Hyper-parameter	Value	Description
Max iteration	10000	The maximum number of iterations per episode.
Episodes	3000	The number of episodes trained for each scenario.
Batch size	256	The batch size for DQN training.
Replay memory size	20000	The capacity of the replay buffer.
Discount factor	0.9	The discount factor γ used in long-term rewards.
Learning rate	0.0025	The learning rate of the neural network.
Softly update weight	0.01	The weight for softly updating the target network.

Additionally, to validate the scalability of the method, we constructed test environments with different network scales. Specifically, we built test scenarios with 50 and 100 hosts, named Scenario 2 and Scenario 3, respectively. The number of sensitive hosts remains unchanged at three. Therefore, as the network scale expands, the rewards become sparser.

### Result discussion

(1) Performance comparison under different algorithms (for **RQ1**)

**Fig 5:**
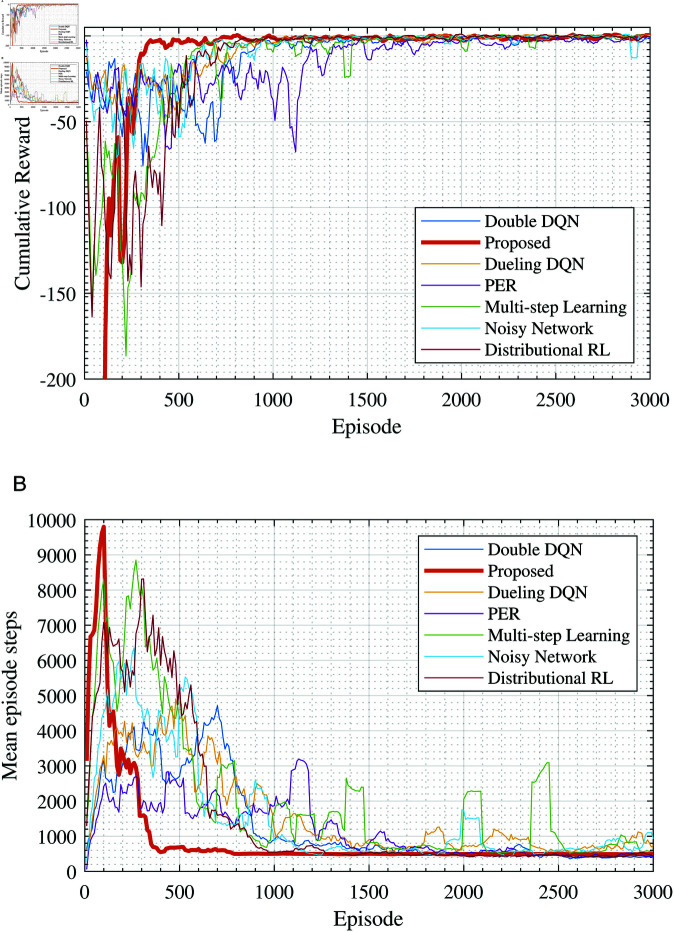
Comparison of training results of different deep learning algorithms (Scenario 1). (a) Cumulative rewards during the training process, (b) Mean episode steps during the training process.

The learning objective for the Agent is to acquire access to all sensitive hosts within the target environment using fewer steps, thereby obtaining a reward value. Thus, the reward value can be used as a measure of the agent’s policy performance. Fig 5 illustrates the training performance under different learning algorithms, where Fig 5(a) shows the change in cumulative reward per episode over the number of training episodes, and Fig 5(b) shows the change in the number of steps per episode over the training steps. From Fig 5(a), it can be observed that during the initial training phase, the rewards obtained by the agent per episode are relatively small, but these increase as training progresses.

Comparing the training processes of different algorithms, it can be seen that during the initial exploration phase, Noisy Network, Double DQN, Dueling DQN, etc., maintain a relatively low “negative reward”, with the rewards per episode ranging between –25 and –50. However, they require approximately 800–900 episodes to converge. As training continues, the convergence speed of different algorithms becomes apparent. The proposed algorithm in this paper converges to the maximum cumulative reward value in approximately 350 episodes, while other algorithms eventually converge after more than 500 episodes, with the longest taking up to 1300 episodes. Regarding the change in the number of steps per training episode, as shown in the results from Fig 5(b), the proposed method converges faster and more stably compared to other algorithms. Compared with other algorithms, the proposed Double DQNchieves convergence nearly 500 episodes earlier.

From the experimental results, it can be seen that the learning curve of the proposed method rises rapidly and quickly approaches the optimal value, maintaining a high level of average cumulative reward throughout the training process. This indicates that the method can effectively utilize training data to accelerate convergence speed. By integrating various DQN improvement techniques, it contributes to a more stable learning process, effectively reducing estimation errors and variances, thereby enhancing the robustness of the policy. The main reason lies in that these improvement techniques address certain limitations of DQN to some extent; for instance, Multi-step Learning and Distributional RL primarily focus on multi-step returns and reward distributions, providing richer learning information, thus their learning curves rise faster. Dueling DQN and Double DQN mainly concentrate on evaluating action values and reducing overestimation, hence they achieve relatively higher learning rewards. The experimental curves of these methods in Fig 5(a) also verify these viewpoints. Therefore, after integrating different DQN improvement techniques, the proposed method demonstrates good performance.

**Fig 6:**
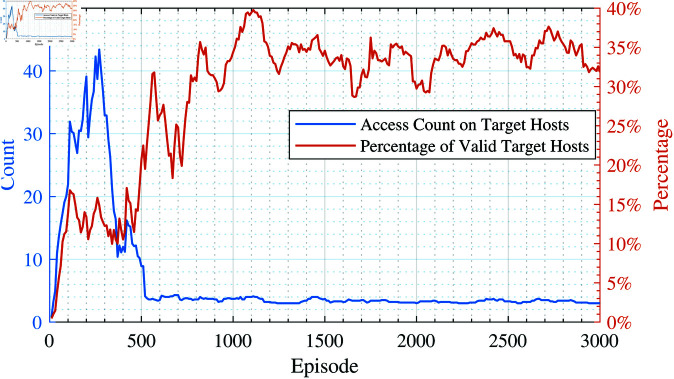
Number of accesses to the target hosts per episode and the proportion of accesses to the target hosts.

During the aforementioned experiment, we recorded the proportion of accesses to the target host by the proposed method, with the results shown in Fig 6. As can be seen from Fig 6, in the early stages of the experiment, the agent accessed the target hosts with a probability of less than 20%, indicating that the agent spent considerable time attempting accesses on other hosts. However, after fewer than 500 episodes, the proposed method could identify the target host more accurately for penetration testing, increasing the proportion of accesses to the target hosts to between 30% and 40%.

**Fig 7:**
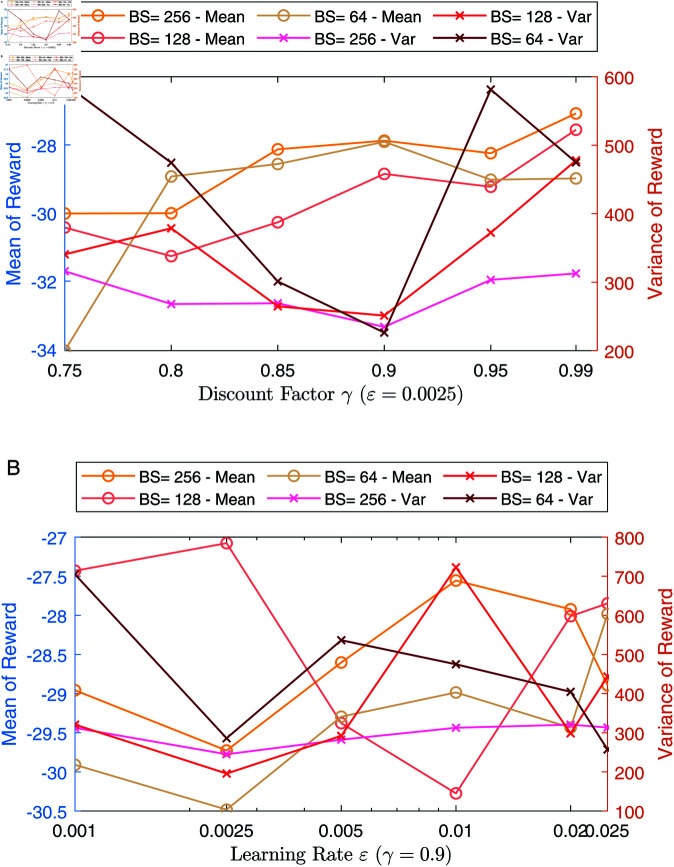
Effect of hyper-parameters on mean and variance of rewards in the first 100 episodes. (a) Results on different discount factors and batch sizes, (b) Results on different learning rates and batch sizes.

Fig 7 discusses the impact of hyper-parameters on model performance, with Fig 7(a) showing results under different discount factors and Fig 7(b) under different learning rates. We evaluated performance using the mean and variance of rewards from the first 100 episodes. The experimental results indicate that while the changes in the mean reward across different hyper-parameters are not particularly significant, staying within a range of –34 to –26, the variations in reward values are more noticeable. Specifically, for Fig 7(a), when the learning rate of 0.0025, the model performs best at a discount factor of 0.9 (with an overall smaller variance), especially with a batch size of 256, leading to a relatively stable variance curve. In Fig 7(b), with a discount factor of 0.9, the model shows its best performance.

(2) Performance comparison in different network scales (for **RQ2**)

**Fig 8:**
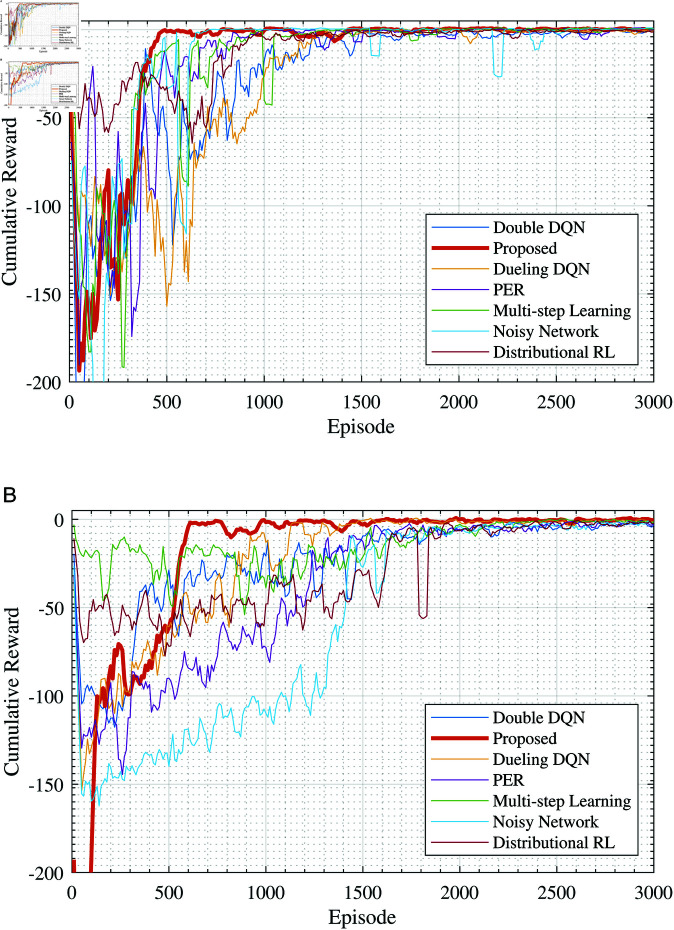
Comparison of training results under different network scales. (a) Cumulative rewards (Scenario 2), (b) Cumulative rewards (Scenario 3).

Fig 8 presents the training curves of several DQN algorithms under two experimental scenarios: one with 50 hosts and another with 100 hosts. It can be observed that as the scale of hosts in the network increases, more episodes are required for various algorithms to converge. Especially when the number of hosts in the network increases to 100, due to the rewards becoming increasingly sparse (the proportion of sensitive hosts is only 2%), the performance differences exhibited by different models become more pronounced. Under the experimental scenarios constructed in this study, the performance of Noisy Network is the worst, followed by Distributional RL; whereas Double DQN and Dueling DQN perform relatively better. The proposed method in this paper converges around episode 500 in scenario 2 and around episode 600 in scenario 3, showing a faster convergence rate compared to other algorithms.

Additionally, we further quantitatively discussed the performance metrics of the aforementioned methods under different network scales: average reward over the first 10 episodes, average steps over the first 10 episodes, and the episode number when the cumulative reward first becomes non-negative. The results are shown in Table 5. It can be seen from the results that the proposed method shows a significant improvement in the episode number when the cumulative reward first becomes non-negative compared to other methods. In scenario 1, the proposed method converges at the $350^{\text{th}}$ episode. In contrast, the fastest among other methods requires 550 episodes (the method based on Noisy Network), and the worst performer is the method based on Double DON, which requires 1200 episodes. When the network scale expands to 50 and 100 nodes, the proposed method converges at the $460^{\text{th}}$ and $610^{\text{th}}$ episodes, respectively. These results also indicate that reinforcement learning-based methods have certain scalability, capable of adapting to larger network scale testing scenarios. Moreover, after integrating various improved strategies and prior knowledge, deep reinforcement learning-based methods can complete testing tasks more quickly. However, the proposed method’s performance in the initial stage is somewhat inferior to other methods. For example, in scenario 1, the average cumulative reward of the first 10 episodes is only −237.70, whereas Dueling DQN achieves −28.30. The proposed method requires 6672 steps in the first 10 episodes, while PER needs only 1993 steps. This result can be understood to some extent as the proposed method focusing on exploring new knowledge rather than just immediate rewards during the initial stage. This contributes to a comprehensive understanding of the system beforehand, improving subsequent execution efficiency, thereby enabling the method to complete the testing work more quickly.

**Table 5:** Performance metrics under different scenarios.

Metric Name	Method	Scenario 1	Scenario 2	Scenario 3
Average Reward over the First 10 Episodes	Double DQN	−31.85	−149.78	−269.85
	Dueling DQN	−25.18	−94.85	−119.34
	PER	−28.23	−92.55	−127.19
	Multi-Step Learning	−110.68	−129.60	−147.85
	Noisy Network	−34.55	−117.33	−131.93
	Distributional RL	−93.10	−138.53	−151.78
	Proposed	−237.70	−279.18	−315.18
Average Steps over the First 10 Episodes	Double DQN	2713	6132	7772
	Dueling DQN	2935	4152	6120
	PER	1993	4578	6938
	Multi-Step Learning	4572	5152	8814
	Noisy Network	4280	5320	9274
	Distributional RL	4389	5311	7152
	Proposed	6672	7880	9794
Episode of the First Non-negative Cumulative Rewards	Double DQN	1200	1250	1540
	Dueling DQN	560	790	1130
	PER	840	910	1560
	Multi-Step Learning	640	960	1800
	Noisy Network	550	830	1750
	Distributional RL	810	1280	1740
	Proposed	350	460	610

## Conclusion

Penetration testing, as a critical approach to information security protection, enhances the cybersecurity defenses capabilities of power IoT systems. In this paper, we introduce an automated penetration testing method based on deep RL technology. This work consists of two parts: a state space construction method that integrates prior knowledge such as system and security knowledge, and a training method based on attention mechanisms and Rainbow DQN. Through the establishment of a simulated testing environment, the effectiveness of the proposed method was verified. Experimental results indicate that the proposed method significantly improves the learning efficiency of the agent during the training process and can adapt to networks of varying scales.

However, limitations remain in this work. For instance, only a limited number of vulnerabilities were injected into the target environment, which may differ from real-world scenarios. Therefore, we plan to continuously update datasets to cover more types of attack methods, and enhance the adaptability of the proposed method in complex scenarios by constructing more sophisticated adversarial simulation environments and participating in cybersecurity competitions. Additionally, although experiments considered networks of different sizes, it remains to be confirmed whether the proposed method applies to realistic or larger scale environments. Integrating the module libraries provided by traditional tools as the foundation for the action space of deep learning agents, and deploying a hierarchical structure of agents is expected to enhance testing efficiency and accuracy. Looking forward, we will continue to explore this field and strive to apply our findings to the real world.

## Supporting information

S1 File Appendices (ZIP)

## Nomenclature

Symbol: Definition
𝒮: State space in reinforcement learning
𝒜: Action space in reinforcement learning
ℛ: Reward function in reinforcement learning
ℱ: State transition probability function
Π: Set of Policies
$s_{t}\in 𝒮$: Agent observes the state from the environment at each time step *t*
$a_{t}\in 𝒜$: A selected action according to a policy in π at each time step *t*
$r_{t}\in ℛ$: The immediate reward from the environment at each time step *t*
$Q(s_{t},a_{t})$: Q-function (i.e. The expected return for taking action *a*_*t*_ in state *s*_*t*_)
γ: The discount factor
ε: The learning rate
θ: The neural network parameters
ℒ: The loss function for Deep Q-Networks (DQN)
*V*(*s*): The value stream in Dueling DQN
*A*(*s*): The advantage stream in Dueling DQN
$\chi_{i,t}$: A weight in an attention network
x: The connection relationship of a host node
$\ell_{i,t}$: The inner product of automatically learned parameters ${𝐖}_{k}$ and ${𝐖}_{q}$
$\omega_{i}$: The weight in the neural network
$\mu_{\omega_{i}}$: The mean value of the weight
$\sigma_{\omega_{i}}$: The standard deviation of the weight
η: A noise vector following a certain distribution
Z(s,a): A probability distribution describing all possible cumulative rewards
*T*_*z*_: A new distribution to be found in DQN
*T*_*p*_: A computed new distribution
*M*_*i*,*j*_: The relationship between the nodes *i* and *j* in the environment
*d*_*m*_: The $m^{\text{th}}$ asset attribute of a node in the environment
*Ass*(*M*_*i*_): The asset attribute vector of the node *i*
*c*_*n*_: The $n^{\text{th}}$ vulnerability attribute of a node in the environment
*Attr*(*M*_*i*_): The vulnerability attribute vector of the node *i*
*f*_*t*_: A weighted sum of the relationship obtained from the environment
Θ: The Dueling Network
